# Trajectories and the influencing factors of behavior problems in preschool children: a longitudinal study in Guangzhou, China

**DOI:** 10.1186/s12888-016-0864-z

**Published:** 2016-06-01

**Authors:** Peng Bao, Jin Jing, Yu Jin, Xumin Hu, Buyun Liu, Min Hu

**Affiliations:** Department of maternal and child health, School of Public Health, Sun Yat-Sen University, Guangzhou, China; Sun Yat-Sen Memorial Hospital, Guangzhou, China

**Keywords:** Preschool children, Behavioral problems, Longitudinal research

## Abstract

**Background:**

Since child mental health problem was a global health issue, many researchers in western countries has focused on the trajectory of it to provide evidence for prevention programs. We designed this study to determine the trajectories of children’s behavior problems, and to explore the effect of parent predictors on children’s behavior problems in Guangzhou, China.

**Methods:**

Children (*N* = 1480) for this longitudinal, population-based survey, were recruited from eight regular kindergartens (October, 2010) across four districts in Guangzhou. Repeated measurement design analysis was used to compare the variation in behavioral problems by gender, only child status, and temperament. Logistic regression was applied to analyze the effect of parents’ risks (maternal depression, parenting style) on the change in child problem behaviors.

**Results:**

The scores of behavior problems (externalizing, emotional, social communication problems) were stable during the entire preschool period by gender and child number. Children with difficult temperament exhibited more problem behaviors than children with easy temperament in the early years, and the misbehaviors declined significantly over time. Moreover, maternal depression and the increase in excessive interference/over protective or punishing parenting strategies resulted in an increase in child behavior problems.

**Conclusion:**

There was no difference between the only-child status and child with siblings in the trajectory of problem behaviors. Parent factors were significant predictions of trajectory of child behavior problem during preschool age.

## Background

The preschool period is important for brain and self-regulation skill in children. Mental health symptoms, beginning in the preschool period, frequently impede the completion of adolescent and adult life tasks [1]. The sequelae include friendship and learning difficulties, school dropout, substance abuse, family violence, and suicide [2]. Mental health problems primarily consist of externalizing (aggression, oppositional defiance, attention deficit, and hyperactive disorder) and internalizing (anxiety and depression) behavior problems [3], and affect proximately 20 % of children in modern societies [4–6]. Moreover, the preschool period is a critical period for the development of social skills, especially the theory of mind. The child social communication problems, such as peer relationships, popularity and pro-social behaviors can be recognized in this period. They may result from externalizing or internalizing problems, or might have been originally present. In the recent years, social communication problems have gradually become the third most common behavior problem [7].

Over the past decade, substantial researches in the western countries have pointed to the prevalence and trajectory of behavior problems in preschool and school age children. In general, the decline in externalizing behavior problems and the relatively stable rate in internalizing behavior problems were in common during the preschool age, either in community or population-based studies [8, 9]. However, the results showed significant diversity in the different social-cultural environment. A comparative study of behavioral problems between China and Germany showed that Chinese children had more emotional and behavioral problems than German children, especially internalizing problems among girls [10]. The research in Shenzhen, China has shown a lower prevalence in emotional problems in 6-year old than in 3-year old children [11], while another investigation in Guiyang indicated a higher rate of behavior problems in 6-year old as opposed to 3–5 year old children [12]. However, the studies in China were all cross sectional ones. As far as we know, no longitudinal study has been conducted in China to report the trajectory of children’s behavior problems.

Another special social factor that may influence the behavior and developmental patterns of children in China is the ‘one child’ policy. There are over 80 % children who are the only child in their families in China. These children have much less social interaction with peers than children have siblings, which cannot be replaced by parents [13]. Earlier studies from other countries indicated that the only children are often overprotected and self-centered, which may have a negative effect on their psychological development [14–16], and especially on the externalizing problems [17]. However, in Bayer’s study, the absence of older siblings was a key predictor of internalizing problems [18]. Research in Japan has also revealed that children with a larger number of older brothers/sisters had a smaller incidence of problematic behaviors [19]^.^ On the other hand, the cross-sectional research in China has found that the children with siblings had worse mental health based on the resource dilution theory which asserts that family resources are divided by the number of children [20]. Although there were many studies focused on the only children, there is no population-based literature that dealt with the trajectory of their behavior problems.

According to Bronfenbrenner’s ecological systems theory, experiences within the family, which is the main social contextual system for the preschool period, influence the developmental trajectories of children’s behavioral orientations [21, 22]. Accumulated evidence indicates a number of risks associated with children’s externalizing and internalizing difficulties. Generally, child risks include physical health problems and difficult temperament [1, 8, 23, 24], while family risks include time-invariant factors, such as young and poorly educated parents, divorced family, low-income, and time-varying factors, such as maternal depression, controlling parents, lack of warmth/sensitivity, overprotective parenting interactions and harsh discipline. Although these risk factors influence the occurrence or continuity of children’s behavior problems, the trajectories of parents’ factors, especially maternal depression and parenting styles were most direct and important ones [24–27]. A deep understanding of parents’ factors effect on trajectory of child problem behaviors, which can support the intervention project, is still required in China.

Therefore, the main purpose of this study was to investigate the trajectories of preschool child behavior problems, especially those of only children, based on a population sample from Guangzhou, China. An additional goal was to explore how maternal depression and parenting strategies predict trajectory of child behavior problems in this sample.

## Methods

### Sampling methods and study population

This study was a prospective investigation with a stratified cluster sampling method. The data were collected in three waves (Oct, 2010, Nov, 2011, and Jun, 2013). The vast majority of children (3–6 years) attended a kindergarten near their home in China. The families were recruited at eight kindergartens in two of eight Guangzhou districts with a medium economic level. 2258 parents of 3–4-year-old children (healthy babies at birth, not adopted) were enrolled in the study and 2048 participants were willing to participate in the research. In wave 2 investigation, 1747 participants completely finished the questionnaires, while 1480 (87.4 %) participants were retained for the final investigation in 2013. The mean age of the children was 3.65 ± 0.59 years at the time of recruitment.

The reasons for participants’ exclusion in wave 2 and 3 were moving from the area, the inability to contact them after the initial investigations, loss of interest in the study, other duties, poor physical condition of the child, or missing over 5 % of independent variables in the questionnaire. The socio-demographic characteristics and the mean score of behavior problems in wave 1 for the missing people did not significantly differ from those for the final participants. This implied that the participants included in the analyses were broadly representative of those originally recruited.

### The questionnaires

#### Socio-demographic characteristics

Based on the research of the interaction between the socioeconomic context and human development [28], we designed a questionnaire based on the socioeconomic and family background. The questionnaire included four questions including the parental childbearing age, education level, the family’s monthly income per capita and the family structure. The parental education level was divided between less than or over 12 years, which presented that whether they finished formal education (secondary school). The family’s monthly income per capita was classified into less than or over 5000 China Yuan (CNY), which corresponds to the average income level of Guangzhou families. The family structure was categorized as the nuclear family, extended family and single-parent family.

#### Behavior problems

The instrument used to assess the child behavior problems was a parent-report version of the Strengths and Difficulties Questionnaire (SDQ). The questionnaire included 25 items on three-point Likert scales, which is widely used in screening behavior problems. This method has demonstrated acceptable levels of reliability and validity in Chinese children [29]. The SDQ items were divided into five subscales: emotional symptoms, conduct problems, hyperactivity/inattention problems, peer relationship problems, and pro-social behavior, with five items in each domain. The first four of these subscales could be combined to a total difficulty score [30]. In this study, we also combined conduct problems and hyperactivity problems, as externalizing problems, and peer-relationship problems and pro-social behavior problem (reverse scoring), as social interaction problems. The repeated reliability (the test-retest reliability) of each domain in this study was between 0.45–0.80.

#### Child temperament

The Behavioral Style Questionnaire (BSQ) included 100 items, with a six-level scoring system [31]. The items presented nine dimensions of child temperament: rhythmicity, activity level, approach/withdrawal, adaptability, reaction intensity, mood quality, persistence, distractibility, and sensory threshold. The items were averaged to specify a global measure of child temperament as easy, difficult, or slow to warm-up. The slow to warm-up and difficult temperament types both have similar evaluation standards, e.g. both of them are characterized by low rhythmicity, withdrawal in a new environment, poor adaptability, and negative emotions [31]. Moreover, lots of studies in China indicate that children with difficult and slow to warm-up temperaments exhibited no significant difference in behavior problems, and that both had more behavioral problems than the easy temperament children [32, 33]. Therefore, we combined the slow to warm-up into difficult temperament children in our analyses. The repeated reliability of each domain in this study was between 0.57 and 0.85.

#### Maternal depression

Maternal depression was measured by the standardized Center for Epidemiologic Studies Depression Inventory (CES-D) [34]. This is a 20-item, self-report checklist that assesses the adult depressive symptoms. The repeated reliability in this study was 0.74.

#### Parenting style

We used the Egma Minnenav Bardndosna Uppforstran (EMBU), revised in Chinese, to assess parenting strategies and behaviors [31]. We excluded the subscale of partiality to one child more than the others, and chose the remaining four subscales (emotional warmth/understanding, excessive interference/protection, rejection/denial, and harsh/punishment) to evaluate the parenting strategies. The questions were changed from a child-report to a parent-report, with acceptable validity and reliability [35].

#### Data collection

The children in this study were all a part of large kindergarten classes, with teacher to children ratios below 1:10. Due to the large class sizes, we assumed that the teacher reports of child behavior may be less accurate than their primary caretaker reports. Therefore, we chose the primary taker to be the main respondent for completing the BSQ, SDQ, EMBU, and socio-demographic questionnaires. The primary caretaker was defined as the person with the greatest responsibility for the daily care of the child in the family [26]. In 95 % of cases, the children’s primary care taker was the mother, while in 5 % cases it was either the father or the grandparents.

Wave 1 survey was conducted after receiving written informed consents signed by every child’s parents. BSQ, SDQ, EMBU, and socio-demographic questionnaires were completed in period 1. SDQ and CES-D were completed in wave 2. In the last wave, SDQ, CES-D, and EMBU, questionnaires were completed. The repeated reliability of the questionnaires was tested 4 weeks after the completion of period 1 by 202 parents (10 % of the sample). In wave 1 survey, the questionnaires were sent out and collected in kindergartens by the teacher in charge, with two investigators in each of the participating classes present during parent meetings. In the last two surveys, the questionnaires were distributed among the parents when they picked up their children from kindergarten and were returned 1 week later. The drop-out families were interviewed by telephone or email to inquire about the reason why they discontinued in the study.

#### Statistical analysis

The data was uploaded in Epidata 3.1 software, and the analyses were conducted using Statistical Package for the Social Sciences (SPSS) 22.0. To examine the trajectory of problem behaviors in children, the Repeated Measures Analysis of Variance (RMANOVA) was conducted based on gender, child number (only child or child with siblings), and child temperament (easy or passive). We chose to model a continuous (rather than dichotomized) measure of behavior problems, since a dimensional approach better characterizes the processes across the entire range of functioning in the population and since subclinical difficulties are also associated with impaired functioning and development of clinical disorders [4].

In order to analyze the effect of parent factors (parenting style, and maternal depression) on behavior problems, logistic models were used. Firstly, logistic regressions for each dependent variable, including maternal depression and parenting strategies, were carried out. Variables showing significant contribution to changes of child behavior problem (Wave 1 to Wave 3) were entered into a backward step-wise logistic regression, and the demographic characteristics were included as controlling factors in the adjusted model [36].

To account for missing values in the independent variables in our analyses, maximum likelihood estimating method was employed to reduce the bias in the parameter estimates.

## Results

### Descriptive Statistics of Study Variables

#### Sociodemographic characteristics

As presented in Table 1, sociodemographic characteristics were stable in all three waves. The final sample consisted of: 51.7 % boys, 82.3 % of children the only child, approximately 75 % highly educated parents, and 57.8 % nuclear families. 45.2 % families had lower income than average (RMB 5000).

**Table 1:** Sociodemographic characteristics in every period

Characteristics	Wave 1 n(%) *n*_t_ = 2048	Wave 2 n(%) *n*_t_ = 1747	Wave 3 n(%) *n*_t_ = 1480
Child			
Male	1057(51.6)	891(51.0)	765(51.7)
Only child	1560(82.5)	1431(81.9)	1218(82.3)
Parents			
Father’s age (mean ± SD, range)	33.14 ± 4.14,21-54	34.09 ± 4.17,23-54	35.24 ± 4.19,24-54
Mother’s age(mean ± SD, range)	29.89 ± 3.68,20-52	30.98 ± 3.85,21-50	32.10 ± 3.74,21-49
Father’s education ≥ 12y	1568(77.9)	1378(78.9)	1162(78.5)
Mother’s education ≥ 12y	1506(74.8)	1310(75.0)	1095(74.0)
Family			
Nuclear family	1154(56.9)	984(56.3)	855(57.8)
Income per capita < 5000 CNY	960(46.9)	847(48.5)	669(45.2)

### Child behavior problems, parenting styles, and maternal depression in three waves

The general trajectory of externalizing, emotional, social communication problems, and the total difficulty were stable during the entire preschool period (Table 2).

**Table 2:** Scores of SDQ, EMBU and CES-D in the three waves (Mean ± SD)

	Wave 1	Wave 2	Wave 3
Child Behavior (SDQ)			
Emotional symptoms	1.97 ± 1.62	1.89 ± 1.72	1.95±1.65
Externalizing problems			
Conduct	1.89 ± 1.36	1.71 ± 1.31	1.73 ± 1.41
Hyperactivity	4.10 ± 2.10	4.18 ± 2.16	4.08 ± 2.11
Social communication			
Peer relationship	2.30 ± 1.56	2.11 ± 1.57	2.29 ± 1.55
Prosocial behavior	6.94 ± 1.92	7.21 ± 1.94	7.06 ± 1.94
Total difficulty	10.26 ± 4.53	9.88 ± 4.58	10.07 ± 4.61
Parenting Styles (EMBU)			
Emotional warmth	52.04 ± 8.98	n/a	54.67 ± 10.60
Excessive interference	34.68 ± 6.64	n/a	35.02 ± 6.87
Rejection	13.26 ± 3.46	n/a	11.97 ± 2.81
Punishment	11.45 ± 1.98	n/a	10.83 ± 1.79
Maternal Depression (CES-D)	n/a	18.34 ± 2.58	18.43 ± 2.64

### Trajectory of child problem behaviors

RMANOVA analysis of children’s problem behaviors were preceded by gender, temperament, and only child status.

#### Trajectory difference by gender

**Fig. 1:**
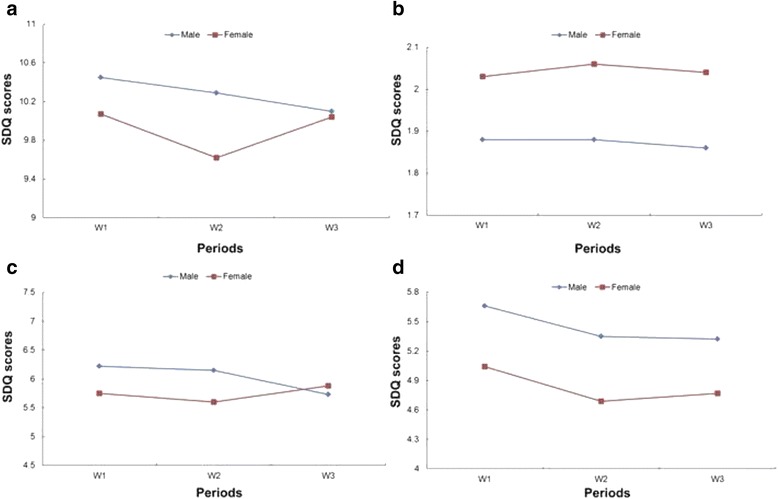
Trajectory of child behavior problems by gender. **a** Total difficulty, mean SDQ scores of boys in three periods: 10.45, 10.29, 10.10; SDQ scores of girls: 10.07, 9.62, 10.04. **b** Emotional problem, SDQ scores of boys: 1.88, 1.88, 1.86; SDQ scores of girls: 2.03, 2.06, 2.04. **c** Externalizing problem, SDQ scores of boys: 6.22, 6.15, 5.73; SDQ scores of girls: 5.75, 5.60, 5.88. **d** Social communication problem, SDQ scores of boys: 5.66, 5.35, 5.32; SDQ scores of girls: 5.04, 4.69, 4.77

The age slopes for boys and girls were examined and compared in the unconditional model (Fig. 1). The data was fitted according to the normal distribution, in the Mauchly sphericity test, P <0.01, with Greenhouse Geisser method chosen for the adjustment. No significant difference was observed in total difficulty, during 3 years (main effect of age: *F* = 0.929, *p* = 0.388). The differences between boys and girls were not significant either (main effect of gender: *F* = 1.650, *p* = 0.199). Therefore, it is important to emphasize the lack of significant difference between both boys and girls in trajectory of total difficulty. For emotional and externalizing problems, no significant difference was observed during the 3 waves in boys or girls (main effect of age—emotional: *F* = 0.039, *p* = 0.955, externalizing: *F* = 0.686, *p* = 0.482; main effect of gender—emotional: *F* = 3.188, *p* = 0.075, externalizing: *F* = 2.556, *p* = 0.110; interaction effect of age and gender—emotional: *F* = 0.020, *p* = 0.976, externalizing: *F* = 3.016, *p* = 0.058). In addition, child social problem behaviors exhibited a declining trajectory in this period (main effect of age: *F* = 3.398, *p* = 0.037), while in boys it declined more than in girls (main effect of gender: *F* = 12.801, *p* < 0.001). There was no significant difference between the interactive effect between age and gender (*F* = 0.073, *p* = 0.919).

#### Trajectory difference by child number

**Fig. 2:**
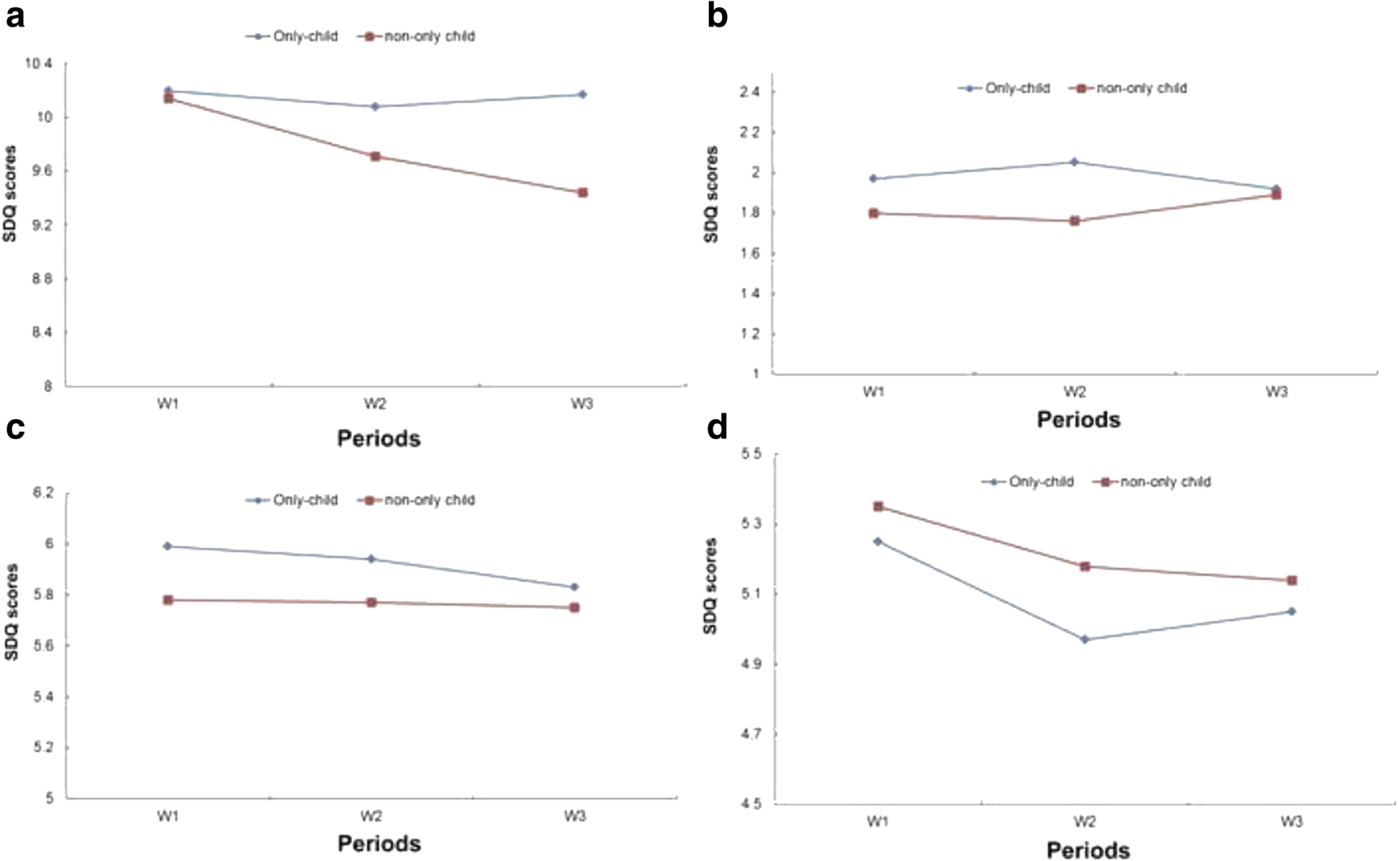
Trajectory of child behavior problems with respect to the number of children. **a** Total difficulty, mean SDQ scores of the only-child in three periods: 10.20, 10.08, 10.17; SDQ scores of non-only child: 10.14, 9.71, 9.44. **b** Emotional problem, SDQ scores of the only-child: 1.97, 2.05, 1.92; SDQ scores of non-only child: 1.80, 1.76, 1.89. **c** Externalizing problem, SDQ scores of the only-child: 5.99, 5.94, 5.83; SDQ scores of non-only child: 5.78, 5.77, 5.75. **d** Social communication problem, SDQ scores of the only-child: 5.25, 4.97, 5.05; SDQ scores of non-only child: 5.35, 5.18, 5.14

The data was fitted according to normal distribution. The Mauchly sphericity test showed P <0.01, while the Greenhouse Geisser method was used for the adjustment. The data indicated no significant difference between the only or non-only child in total difficulty, emotional, externalizing and social communication problems during these 3 years (main effect of only child: *F* = 0.888, *p* = 0.347; *F* = 1.481, *p* = 0.224; *F* = 0.341, *p* = 0.559; *F* = 0.297, *p* = 0.586; interaction effect between age and number of children: *F* = 0.577, *p* = 0.544; *F* = 0.535, *p* = 0.575, *F* = 0.033, *p* = 0.949, *F* = 0.055, *p* = 0.936) (Fig. 2).

#### Trajectory difference by child temperament

**Fig. 3:**
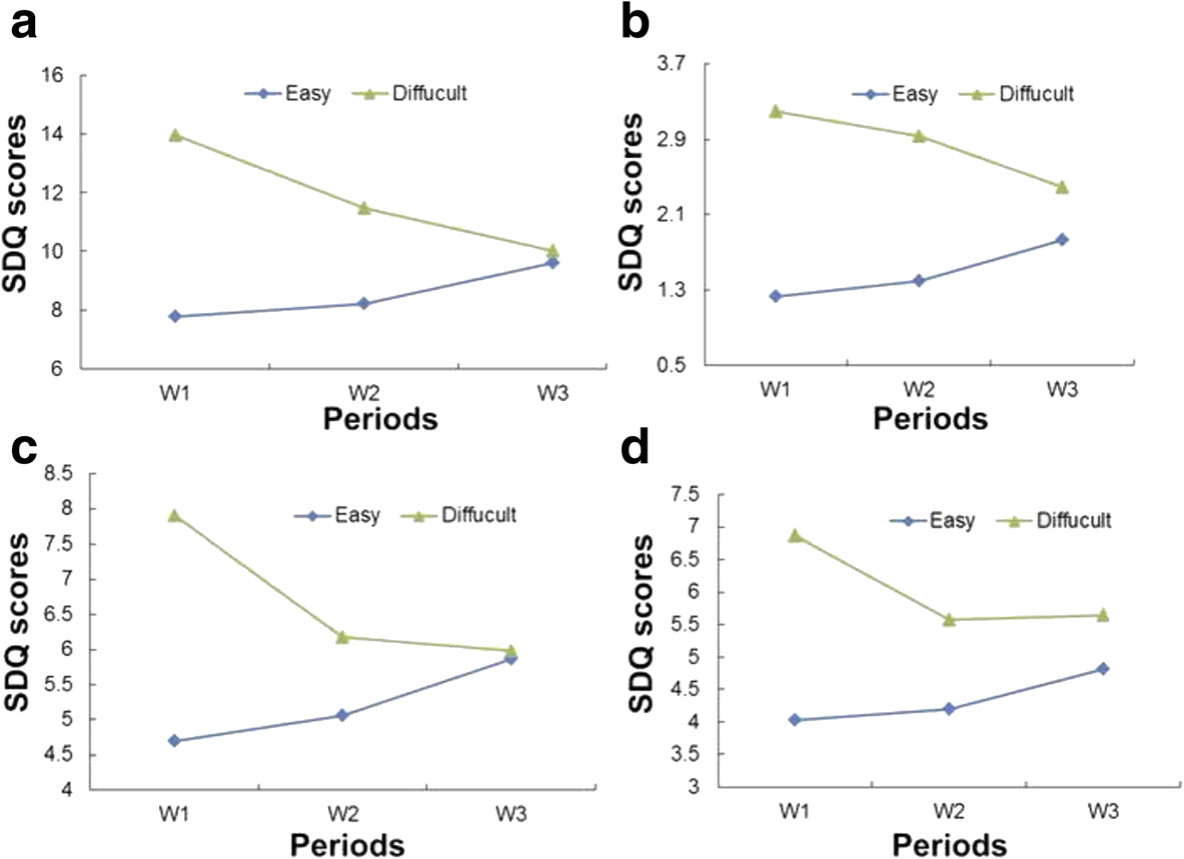
Trajectory of child behavior problems by child temperament. **a** Total difficulty, mean SDQ scores of easy child: 7.76, 8.19, 9.62; SDQ scores of difficult child: 13.98, 11.47, 10.02. **b** Emotional problem, SDQ scores of easy child: 1.23, 1.39, 1.83; SDQ scores of difficult child: 3.20, 2.93, 2.39. **c** Externalizing problem, SDQ scores of easy child: 4.69, 5.06, 5.86; SDQ scores of difficult child: 7.91, 6.17, 5.98. **d** Social communication problem, SDQ scores of easy child: 4.02, 4.20, 4.81; SDQ scores of difficult child: 6.87, 5.57, 5.64

The data was fitted according to normal distribution and spherical symmetry. Children with easy or difficult temperament presented significantly different trajectories for each of the problem behaviors (main effects of child temperament: total difficulty: *F* = 36.750, *p* = 0.000; emotional: *F* = 34.600, *p* = 0.000; externalizing: *F* = 16.142, *p* = 0.000; social communication: *F* = 28.995, *p* = 0.000). Difficult temperament in children (3–4 years old) in wave 1 predicted more problem behaviors than easy temperament. Children with easy temperament maintained almost the same score of problem behaviors from preschool period until 6–7 years (wave 3). However, problem behaviors of children with difficult temperament declined substantially and no significant difference was observed in comparison to children with easy temperament in 6–7 years old. Moreover, problem behaviors declined more sharply in the early (W 1 to W 2) compared to the late preschool period (W 2 to W 3) (main effects of time: *F* = 13.669, *p* = 0.000; *F* = 5.593, *p* = 0.004; *F* = 8.699, *p* = 0.000; *F* = 9.129, *p* = 0.000; interaction effect of age and child temperament: *F* = 14.344, *p* = 0.000; *F* = 7.841, *p* = 0.000; *F* = 11.775, *p* = 0.000; *F* = 8.259, *p* = 0.000, respectively) (Fig. 3).

### Factors of parents predicted the change of child behavior problem

In unadjusted analysis (Table 3), most factors, excluded maternal depression change from wave 1 to wave 3 and rejection parenting, were predictors of change of child behavior problems. Although emotional warmth and punishment parenting were not significant predictors, they were included in the adjusted model.

**Table 3:** Prediction of change of child behavior problem from wave 1 to wave 3

Potential Predictors	Unadjusted Results^a^		Adjusted Results^a^	
	OR (95 % CI)	*P*	OR (95 % CI)	*P*
Child behavior problem (SDQ) in Wave 1	0.745(0.703–0.790)	<0.001	0.709(0.661–0.760)	<0.001
Maternal Depression (CES-D)				
Wave 1	1.087(1.015–1.165)	0.017	1.133(1.036–1.239)	0.005
Change from wave 1 to wave 3	0.955(0.903–1.011)	0.111	-	-
Change of Parenting strategies from wave 2 to wave 3 (EMBU)				
Emotional warmth	0.983(0.966–1.000)	0.054	0.976(0.954–0.998)	0.030
Excessive interference	1.019(0.998–1.041)	0.075	1.024(0.998–1.052)	0.069
Rejection	0.991(0.948–1.036)	0.697	-	-
Punishment	1.071(0.992–1.155)	0.078	1.124(1.022–1.237)	0.016

^**a**^Sample size was 1480

In the multilevel analysis, the model explained 41 % of variations. Child behavior problems in wave 1 and increase of emotional warmth/understanding parenting were factors that related to decrease of child behavior problems (OR < 1). Meanwhile, maternal depression in wave 1 and increase of punishment predicted the increase of child behavior problems significantly (OR > 1). Excessive interferencing parenting was also in the adjusted model of effect on increase of child behavior problems although not significantly (*p* = 0.069).

## Discussion

### The trajectories of child problem behaviors

According to care taker’s report, children’s externalizing, internalizing, and social communication problems were stabilized during the preschool period. The results were consistent with those obtained from cross-sectional research with similar age groups in Guangzhou or the surrounding cities [37–39].

Firstly, we did not find gender differences in externalizing (Fig. 1b), emotional (Fig. 1c) or social communication problems (Fig. 1d) in this population sample. Some studies from other countries reported a steep decline in externalizing problems during the preschool period, with boys exhibiting more aggressive behavior than girls [9, 40]. The others concluded that the children were already at their peak levels of disruptive behavior when they enrolled in school [41]. While searching the studies in China, we ran into similar results that demonstrated invariance of externalizing problems in both boys and girls [38, 42]. We assumed that the difference in the results from these studies could be attributed to research methodology or a distinction between children themselves in different countries and cultural environments [43]. Similarly, with externalizing problem, the trajectory of emotional problems presented the same stable trend in both boys and girls, which was consistent with other research findings [1, 44]. Girls exhibited higher internalizing problems than boys, but the difference was not significant in our study. The finding agreed with the mainstream view that gender difference in internalizing problems becomes gradually magnified from the early childhood to adolescence [45]. The third type of problem behaviors we focused on were social communication problems, with a significantly declining trajectory in both boys and girls. This result was in line with the development of child’s social skills, which showed rapid improvement during the preschool period [46]. Children (3–4-years-old) exhibited a tendency for sharing toys, being friendly during play-time and felt content. Children 5–6 years old exhibited more helpful behaviors [46]. The boys exhibited more problems than girls, which was attributed to the later language development, more externalizing behaviors that influence their peer relationships, or higher morbidity of social communication problems [7, 46].

In this study, 82.3 % of the participants were the only children, which is representative of the current situation in China. Certain previous studies indicated that the only child was thought to be overprotected, self-centered and lacking peer interaction before the school age, which may aggravate the problem [14–16, 18]. However, no difference in the trajectory of behavior problems was observed between the only or non-only child in this study, which was consistent with other studies in China [20, 38]. The reasons behind the different results between China and the other countries, we assumed, were partially attributed to the Chinese one-child policy. The one child policy was implemented by a series of provisions, for example, families with two or more children would suffer financial punishment while the only child family would be encouraged by obtaining more financial aid [47]. This could result in an economic burden on families with more than one child, and, furthermore, decrease the economic resources available to each child. In addition, in comparison with children with siblings, the only child would obtain more interpersonal resources from the parents, such as attention, time and energy, which may be conductive to their well-being [20, 48]. Lastly, the participants in this study were all enrolled in kindergartens, therefore, the children could learn the skills of cooperation, emotional control, compromise, and understanding from their interaction with peers in class who played the role of siblings. Overall, although the only children could have some negative characteristics in their behavior, they also had more economical and interpersonal family resources available to them, especially due to the only child policy in China, which played a positive role on their behavior [48].

Child temperament is the third factor that has been found to predict child problem behaviors. Children who had more difficult temperaments as infants or toddlers have been found to have more externalizing and internalizing behavior problems during childhood [40, 49]. Miner’s research reported similar findings, that externalizing behavior trajectory declined more steeply for children exhibiting with difficult temperaments early in life [24]. We extended this conclusion to emotional and social communication problems as well. In 6-year-old children, there was no perceived difference in problem behaviors between the children with difficult and easy temperaments, in comparison to the infant phase. This sharp decline may reflect the fact that children with difficult temperaments exhibited more frustration, anger, withdrawal and anxiety at home early on, but over time, became more aligned with peers after they enrolled in kindergarten. Another study reported that this difference in trajectory of temperament was possibly due to a biased report by the parents, the evaluation of which was influenced by the early months dealing with an irritable infant [24].

### Parent effect on change of child problem behaviors

Sufficient literature has documented that family time-varying factors, such as maternal depression, harsh parenting and the family environment, contributed to a high risk of occurrence and continuation of child problem behaviors [22, 24, 25, 49–52]. These factors change over time and with their effects on child behaviors. Moreover, the change in these factors was affected by child behavior [44]. Nevertheless, few studies paid attention to the change in risk factors associated with a change in child problem behavior. This study provided a primary analysis of parent risk factors effect change of child behavior and may provide evidence for an intervention program.

Our results on maternal depression showed that children whose mothers had depressive symptoms during the early years, exhibited increased problem behaviors in later years. This result supported the vast majority of previous researches findings that maternal depression has been shown to have deleterious and long-lasting effects on a child’s cognitive and socio-emotional development [53–56].

Parenting factors that influenced the change in child behavior were the trajectory of warmth/understanding as protective factors, and excessive interference/protection, punishment as the risk factor. This result was in line with previous findings from longitudinal researches of other countries and cross-sectional researches in our country [24, 57, 58]. The increase in the positive parenting attitude and a sensitive response to the child could contributed to a secure attachment, which was linked to many positive outcomes in children and a decrease in problem behavior [59, 60]. However, early negative parenting interactions, whether authoritarian or over interferencing/overprotective, could predict adverse outcomes. Jordana K found overinvolved/protective parenting also being important at preschool age [57]. The mechanisms of risk transmission may be that the parents, who interfered too much with the child’s thoughts and behavior, caused an inhibition in child’s self-expression, thereby promoting problem behaviors [57, 61]. In addition, the present findings confirmed data of other researches highlighting hash discipline as key predictor of early childhood externalizing and internalizing symptoms [18].

As prevention of child mental health is a global public health program, researches that attend to suggest the prevention are warranted. The present findings indicated that prevention efforts should focus on parenting strategies and maternal depression, and such programs could begin in preschool period.

### Strengths, Limitations, and Future Directions

The present study provided the first longitudinal data on the development of children’s problem behaviors during the preschool period in China, with mostly only children in the population sample. We applied contemporary statistical techniques of repeated measures to examine the patterns over time. Last but not the least, the parent effect of change in risk factors and child problem behaviors was analyzed instead of focusing on the one-time point status. This finding strongly supported Bronfenbrenner’s ecological systems theory and directly suggested that intervention programs should pay more attention to children with aggravating family risks [21].

Besides these strengths, the findings from this study should be considered with several limitations. Despite the size and diversity of the sample, the study still lacked scattered children not attending kindergarten. Since the exact proportion of children attending kindergarten could not be found in the year book which was published by statistical bureau of Guangzhou, we could only know from our life experience that vast majority of children went to kindergarten at 3-year old in urban area. Therefore, the sample could not be regarded as representative of the general population. Another limitation of the study was single informants of the predictors and independents, which may have resulted in bias in maternal perception [24]. At last, there were still 3.8 % missing data in every questionnaire, which may be the cause of some bias in the study.

## Conclusions

The results of our study provide an important direction for future researches to determine the effect of influencing factors in more comprehensive, nationwide level. Based on this, population level, randomized, controlled trials of intervention studies are required to test the cost-effectiveness of prevention aimed at reducing children’s problem behaviors.

## Abbreviations

BSQ: Behavioral Style Questionnaire
CES-D: Center for Epidemiologic Studies Depression
EMBU: Egma Minnenav Bardndosna Uppforstran
RMANOVA: Repeated Measures Analysis of Variance
SDQ: Strengths and Difficulties Questionnaire
SPSS: Statistical Package for the Social Science
